# Decreased expression of *miR-200a* and *miR-223-3p* in endometriosis during the secretory phase of menstrual cycle: Insights from a case-control study on molecular biomarkers and disease-related infertility

**DOI:** 10.18502/ijrm.v22i12.18066

**Published:** 2025-01-31

**Authors:** Yasaman Nazari Hagh, Mohamadreza Ahmadifard, Sedigheh Esmaelzadeh, Soheila Abbaszadeh, Naser Shokrzadeh

**Affiliations:** ^1^School of Medicine, Shahroud University of Medical Sciences, Shahroud, Iran.; ^2^Department of Medical Genetics, School of Medicine, Babol University of Medical Sciences, Babol, Iran.; ^3^Infertility and Reproductive Health Research Center, Health Research Institute, Babol University of Medical Sciences, Babol, Iran.; ^4^Reproductive Health Research Center, Clinical Research Institute, Urmia University of Medical Sciences, Urmia, Iran.

**Keywords:** Endometriosis, miRNA, Implantation, Signaling pathways, Biomarkers, Infertility, Menstrual cycle.

## Abstract

**Background:**

Endometriosis (EM) is a condition that causes infertility with decreasing uterine receptivity. It is reported that it affects about 20–25% of all infertile women. Some genetic markers play a crucial role in pathogenesis and infertility.

**Objective:**

This study investigates the role of *miR-200a* and *miR-223-3p* in embryo implantation and their association with EM-related infertility.

**Materials and Methods:**

In this case-control study, 36 women who referred to the Center for Research on Reproductive Health and Infertility of Babol University of Medical Sciences and Fatemeh Al-Zahra Infertility Specialized Treatment Center in Babol, Iran between June 2022 and July 2023 were evaluated. Participants were divided into 2 EM and control groups (n = 18/each). Endometrial samples were collected from participants between 17 ${\;}^{\text{th}}$ and 24 ${\;}^{\text{th}}$ days of their menstrual cycle. Histopathological examination (hematoxylin and eosin and periodic acid schiff) was performed to confirm the secretory stage, and *miR-200a* and *miR-223-3p* expressions were analyzed by quantitative reverse transcriptase-polymerase chain reaction.

**Results:**

Histological analysis confirmed that both groups were in the secretory stage. Additionally, miRNA expression results showed a significant decrease in the *miR-200a* and *miR-223-3p* expression levels in EM group compared to control group. The expression level of *miR-223-3p* and *miR-200a* in the eutopic endometrial tissue of women with EM was notably lower than those in the control group.

**Conclusion:**

Our results suggest that *miR-200a* and *miR-223-3p* are involved in the EM pathogenesis, while other genes and signaling pathways are probably involved in the implantation failure.

## 1. Introduction

Endometriosis (EM) is a chronic estrogen-dependent inflammatory disorder that occurs when the endometrium develops beyond the uterine cavity, and it results in pain and infertility. This tissue may manifest on the surface of the ovary, beneath the uterus, inside the pelvic cavity, and on the intestinal wall (1–3). EM affects approximately 25–50% of infertile women, and about 30–50% of women with EM are infertile (4, 5). The disease disrupts progesterone and estrogen signaling pathways, resulting in progesterone resistance and estrogen dominance (6, 7). This hormonal imbalance induces inflammatory reaction and pelvic discomfort in the afflicted individual diminishes the endometrium's acceptability for embryo implantation and may jeopardize implantation via endometrial malfunction (8, 9). The embryo quality and receptivity of the endometrium are 2 critical components of implantation success (10, 11). Thus, the interplay between the expression levels of various molecules involved in endometrial receptivity and ovarian hormonal concentrations is crucial during embryo implantation (12).

For instance, it has recently been proven that microRNAs (miRNAs) play an essential role in embryo implantation and endometrial receptivity (13). The pierces communicating between the endometrium and blastocyst are essential for the implantation stage, and miRNAs are released from both the endometrium and blastocyst, and the gene's signaling undergoes a shift (14, 15). Therefore, it can be said that miRNAs play a key role in implantation, and considering the impacts of these small molecules in the pathophysiology of EM, there is a strong correlation between EM-related infertility and miRNAs (16, 17).

Hence, it is crucial to investigate these miRNAs in individuals, whose implantation process is disrupted, including EM group. By adjusting inflammation, proliferation, and angiogenesis will enable the implantation of endometrial cells in benign areas, where they play a role in the etiology and development of EM. Thus, it is possible to evaluate certain miRNAs as noninvasive indicators in the molecular diagnosis of this disease (18, 19).

Because there have been numerous studies conducted on genes associated with EM, this study investigates the level of expression of *miR-223-3p* and *miR-200a* in the endometrial tissue in the secretory phase of the menstrual cycle in women with EM, compared with healthy women. However, there is a lack of studies focusing on molecular markers during the specific phase of the uterine cycle in relation to EM and these markers were first detected in EM and the implantation stage.

## 2. Materials and Methods

### Collection of samples and grouping

This case-control study was conducted with 36 women referred to the Center for Research on Reproductive Health and Infertility of Babol University of Medical Sciences and Fatemeh Al-Zahra Infertility Specialized Treatment Center in Babol, Iran between June 2022 and July 2023.

### Inclusion and exclusion criteria

The inclusion criteria were as follows, women aged below 38 yr, had a regular menstrual cycle (between 24 and 35 days), clarity about the last menstrual period, and were willing to participate. Women with autoimmune diseases, endometrial hyperplasia, polyps, polycystic ovary syndrome, uterine anomalies, chronic anovulation, bilateral fallopian tube obstruction, a history of fibroids, and unexplained infertility were excluded.

They were divided into 2 groups (n = 18/each), women with (case) and without EM (control). Women in the EM group were diagnosed by surgery, laparoscopy, or laparotomy and histological sample or ultrasound (Esaote mylab40, Esaote, Italy) provided by the consultant during the follicular period of the menstrual cycle. Almost all women with EM were in stages 3 and 4, and none of the individuals used hormonal medications for at least 3 months before the study.

Endometrial samples were obtained using a pipelle between the 17 ${\;}^{\text{th}}$ and 24 ${\;}^{\text{th}}$ days of the cycle, related to the mid-secretory phase. After washing, the samples were separated into 2 separate groups. The formalin-preserved piece was examined histologically to confirm the endometrium secretory phase. The remaining samples were placed in Trizol (Sigma Aldrich, USA) and stored at -80 C.

### Sample size

Based on similar studies and using the above formula, with a 95% confidence interval and 80% test power, the required sample size was calculated to be 18. Consequently, 18 women with EM (case) and 18 women without EM (control) were included in the study. 


$$n=\frac{2{\left(Z_{1-\frac{\alpha }{2}}+Z_{1-\beta }\right)}^{2}S^{2}}{d^{2}}$$

### RNA extraction 

Total RNA was extracted from 30 mg of endometrial tissue using Trizol (Sigma-Aldrich, USA). The tissue was homogenized with Trizol, followed by the addition of 300 µl of chloroform and centrifugation at 12,000 rpm for 10 min at 4 C. The supernatant was collected, washed with isopropanol, and centrifuged. Subsequently, 500 µl of sterile 75% ethanol was added to the pellet, which was stirred for 5 min and then centrifuged twice at 7500 rpm at 4 C. The RNA pellet was dissolved in 50 µl of nuclease-free H_2_O and heated at 65 C for 3 min. RNA concentration was quantified using a NanoDrop spectrophotometer (Thermo Scientific, Wilmington, USA), with an OD260 of 1.8–2.0 indicating high purity. The isolated RNA samples were stored at -80 C for future analysis.

### cDNA synthesis and real-time polymerase chain reaction (RT-PCR)

cDNA was synthesized from total RNA using a Biofact kit (Cat No.BR631_O96, Biofact, Korea) and stem-loop primers as per the manufacturer's protocol. The reaction mixture included 2xRTpermix, RNase-free dH2O, and miRNA-specific stem-loop primers. Reverse transcription was conducted at 37 C for 5 min, followed by 50 C for 30 min and 95 C for 5 min. The synthesized cDNA was stored at -20 C for subsequent PCR use, as detailed in table I.

Real-time PCR was performed using the ABI StepOnePlus system (ABI Co., USA) with SYBR Green PCR Master Mix (Cat No. DQ383_10h, Biofact, Korea), specific forward primers (Table II), and U6 gene as an internal control. All reactions were executed in duplicate. 2 sets of primers were employed: one for cDNA synthesis and another for quantitative real-time PCR based on the stem-loop method (20). The primer design involved creating specific forward primers for each miRNA, such as *miR-200a* and *miR-223-3p*, and a universal reverse primer applicable to all miRNAs except U6. Key design considerations for the universal primer included minimizing binding site overlaps with the genomes of GM crops, ensuring high GC content, and aiming for a melting temperature (Tm) around 60 C. Specific primers used for gene amplification were derived from corresponding sequences, ensuring specificity in Universal Primer Multiplex Real-Time PCR.

### Histological procedures

The samples of uterine endometrium were used to produce slides for histological examinations. The endometrial tissues were fixed in 10% formalin. The samples were embedded in paraffin then using a microtome, tissue templates were sliced into 5-µ-thick sections. Next, the pieces were then put on slides and dried. Finally, the tissues were stained with hematoxylin and eosin (H&E) and periodic acid schiff (PAS) staining according to standard histological techniques.

**Table 1:** miRNA stem-loop primer sequences (5 ' -3 ' ) used to produce cDNA

**Primer name**	**Primer sequence **
**miR-223-3p (STL)**	GTCGTATCCAGTGCAGGGTCCGAGGTATTCGCACTGGATACGACTGGGGTA
**miR-200a (STL)**	GTCGTATCCAGTGCAGGGTCCGAGGTATTCGCACTGGATACGACACATCGT
**U6 (STL)**	GTCGTATCCAGTGCAGGGTCCGAGGTATTCGCACTGGATACGACAAAAATAT
miRNA: microRNA	

**Table 2:** miRNA primer sequences (5 ' -3 ' ) used in quantitative real-time PCR

**Primer name**	**Primer sequence **
**miR-223-3p (Forward)**	GTCAGTTTGTCAAATACCCCAGTC
**miR-200a (Forward)**	GGGTAACACTGTCTGGTAACGAT
**Universal (Reverse)**	GTGCAGGGTCCGAGGT
**U6 (Forward)**	GCTTCGGCAGCACATATACTAAAAT
**U6 (Reverse)**	CGCTTCACGAATTTGCGTGTCAT
miRNA: microRNA, PCR: Polymerase chain reaction	

### Ethical Considerations

This research was approved by the Ethical Committee of Babol University of Medical Sciences, Babol, Iran (Code: IR.MUBABOL.REC.1401.050). Prior to participation, all participants completed a consent form.

### Statistical Analysis

For quantitative variables, *t* tests (U-Mann-Whitney) and the Chi-square test were used to analyze the data. In all tests, the confidence level was 95% and the level of significance was < 5% (p < 0.05); the software equipment used for data analysis was SPSS 25 (IBM, International Business Machines Corp., New Orchard Road Armonk, New York). The demographic features of the groups were shown using mean ± SD. The Kolmogorov-Smirnov test assessed the normality distribution of quantitative data. Using a one-way ANOVA followed by post hoc tests for multiple comparisons, mean difference between groups were evaluated.

## 3. Results

### Demographic data

**Figure 1:**
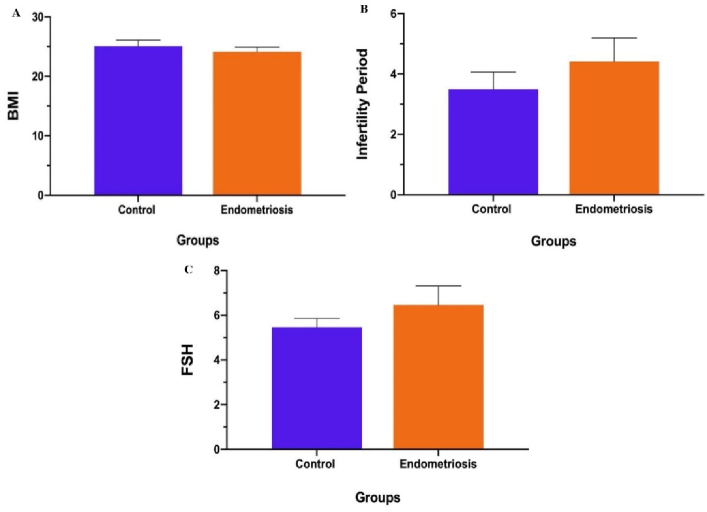
Demographic information in the EM group compared to the control group. A) BMI (p < 0.681), B) Infertility period (p < 0.183), C) FSH (p < 0.296).

The evaluation of demographic data revealed that the body mass index (BMI) in EM group declined slightly, compared to the control group (p = 0.681) (Figure 1).

In contrast, the infertility period and follicle-stimulating hormone (FSH) levels in the EM group enhanced highly, compared to the control group (p = 0.183, 0.296, respectively); however, the difference of variables in both groups was not statistically significant (Table III) (Figure 1).

### *miR-200a* and *miR-223-3p* expressions

**Figure 2:**
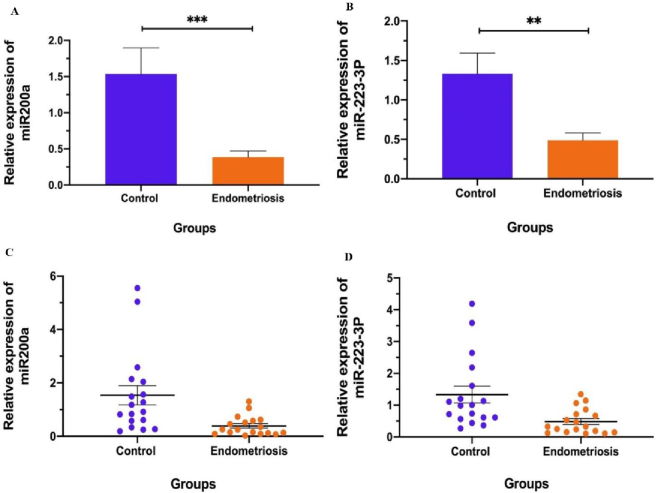
The graph of *miR-200a* and *miR-223-3p* levels in endometrium of EM and control groups via real-time PCR. A) *miR-200a*, B) *miR-223-3p*, C) Scatter plot: *miR-200a* expression in control vs. EM groups, D) Scatter plot: *miR-223-3p* expression in control vs. EM group. **P < 0.001, ***P < 0.0001.

According to the statistical data, the expression level of *miR-200a* (mean ± SD) in the secretory period of the menstrual cycle in the eutopic endometrium of the EM group was significantly reduced than the control group (p < 0.01). Likewise, the expression of *miR-223-3p* was decreased dramatically in the EM group in comparison to the control group (p < 0.01) (Figure 2).

### Histological analysis in the endometrium during the secretory phase

**Figure 3:**
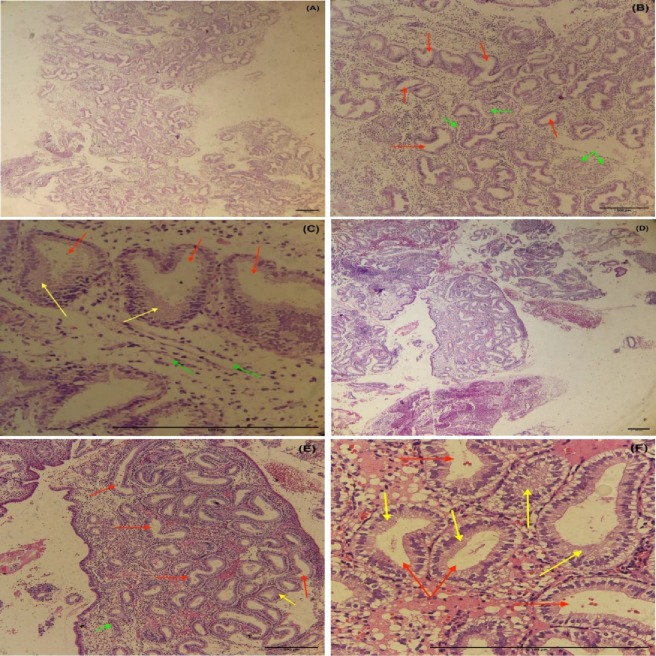
Photomicrograph of uterine endometrial tissue stained with *H&E* in the secretory phase (18 cases in each group). A-C) Control group's endometrial tissue, D-F) EM group's endometrial tissue. Red arrows: Stromal glands, Yellow arrows: Glycoprotein secretions in glands, Green arrows: Vessels.

**Figure 4:**
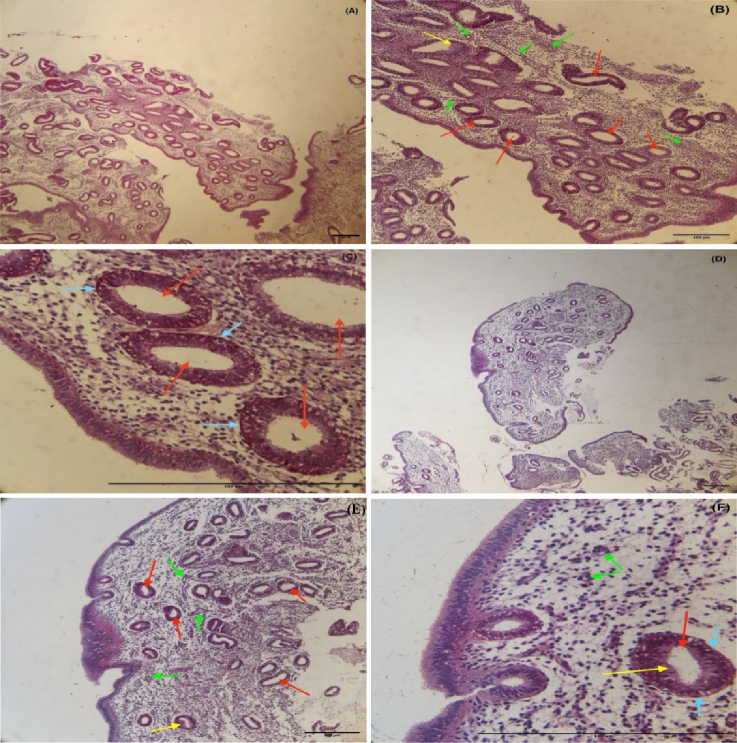
Photomicrograph of uterine endometrial tissue stained with *PAS* in the secretory phase (18 cases in each group). A-C) Control group's endometrial tissue, D-F) EM group's endometrial tissue. Red arrows: Stromal glands, Yellow arrows: Glycoprotein secretions in glands, Green arrows: Vessels, Blue arrows: Basement membrane of glands in the uterine sections.

For the histological investigation, microscopic pictures of the endometrium in the secretory phase of the uterus were captured at x40 magnification. The results of the histological examination in the control and EM group showed that the secretory phase in the menstrual cycle causes changes in the endometrial tissue of the uterus. This includes the stromal glands with glycoprotein secretions, dilated and characterized by cuboidal epithelium. The basement membrane was seen beneath the epithelial cells with low thickness and the number of blood vessels was markedly increased in stroma, verified in the secretory phase of the menstruation (Figures 3, 4).

**Table 3:** Demographic data

**Variables**	**Control**	**EM**	**P-value**
**BMI**	24.66 ± 4.26	24.13 ± 3.28	0.681
**Infertility period**	3.11 ± 2.39	4.47 ± 3.3	0.183
**FSH**	5.45 ± 1.67	6.45 ± 3.63	0.296
Data presented as the Mean ± SD, Chi-squared test. EM: Endometriosis, BMI: Body mass index, FSH: Follicle-stimulating hormone			

## 4. Discussion

EM is a complex condition with various factors contributing to its development. Researchers are exploring potential biomarkers, such as miRNAs, for early detection. miRNAs play a significant role in the implantation process and may impact infertility associated with EM. Additionally, during implantation, miRNAs are released into the bloodstream from the blastocyst and the endometrium. They act on signaling to adjust gene expression. Considering these small molecules' impact on EM's pathogenesis, we can seek an active collaboration between infertility caused by EM and miRNAs. As implantation occurs during the secretory phase of the menstrual cycle, the current study's specimens were likewise collected during this period, and histological methods were used to corroborate the sampling time. The results of histological examination during the secretory phase of the menstrual cycle confirmed sampling time alignment in the study.

The findings of analyzing the expression level of *miR-200a* in the current research indicated that this miRNA had a significant drop in expression in the eutopic endometrial tissue of women with EM compared to the control group (p < 0.000). Research published on the endometrial tissue of EM group also corroborates the reduction of *miR-200a* expression. A survey investigated the expression level of *miR-200a* in EM group -who were in the proliferative and secretory phase of the menstrual cycle and had not received any treatment before sampling- showed decreased expression in ectopic endometrial tissue compared to the eutopic endometrial in these cases (21). Another study investigated the expression levels of *miR-200a* in ectopic and eutopic endometrial of 16 cases with EM. It showed that *miR-200a* had a downregulation in the ectopic endometrial of these cases compared to their eutopic. The women who were in stages 3 and 4 of EM, aged between 24 and 48 yr, and did not receive any hormone treatment 3 months before sampling (22). According to research on the expression profiles of the *miR-200* family throughout the implantation process in mice, the expression level of *miR-200a* was diminished during the preimplantation stage. The *miR-200a* were found to be elevated during decidualization, impacting its target *Zeb1* gene, and *Zeb2* facilitating implantation success (23). Research into the mechanism of miRNAs' action on the target tissue revealed that by targeting the three prime untranslated region of the targeted gene, miRNAs enhance and/or inhibit the expression of such a gene in the organ, consequently influencing the disease process (24). Several potential genes, including *Zeb1* and *Zeb2* (zinc finger E-box binding homeobox 1, 2), were anticipated to have high scores based on target scan searches for *miR-200a* (25). *miR-200a* and its molecular targets, *Zeb1* and *Zeb2*, are regarded as epithelial-mesenchymal transition regulators (EMT) (26, 27). The epithelial-to-mesenchymal transition is a procedure in which epithelial cells lose their polarity and adhesion and obtain invasive and migratory capabilities. Also, they may convert into mesenchymal stem cells, which are capable of transforming into many cell types, in such a manner that they contribute to the beginning and metastasis of cancer development (28). In the substantial percentage of disorders, the expression levels of *miR-200a* and its target genes, especially *Zeb1* and *Zeb2*, had declined, consequently raising EMT activity. In this context, the link between *miR-200a* and its target genes (*Zeb1* and *Zeb2*) in rats with peritoneal fibrosis related to peritoneal dialysis was investigated. *miR-200a* exhibited a drop in expression in these rats, followed by an increase in *Zeb1* and *Zeb2* expression (29). Furthermore, it was shown in a study that increased *miR-200a* expression leads to implantation failure in mice. This investigation was done on female mice aged 6 to 8 wk. Following fertilization, endometrial samples were analyzed. This study discovered that the level of *mmu-miR-200a* expression has decreased at implantation sites. They demonstrated that an increase in *mmu-miR-200a* expression causes implantation failure and a decline in fertility (30). Therefore, the results of the past research compared with results obtained from the present study, it can be concluded that the lowering in the expression of *miR-200a* probably acts by influencing its target genes (*Zeb1* and *Zeb2*). Hence, the upsurge in EMT activity leads to the migration and proliferation of epithelial cells. Since it is essential to suppress the expression of this miRNA for successful implantation, it may be deduced that additional genes and signaling pathways are likely implicated in the failure of implantation in these individuals.

Based on our findings, the level of *miR-223-3p* expression in eutopic endometrial tissue of EM group was considerably lower than in the control group (p < 0.0001). Our study was in line with the findings of research that investigated the degree of *miR-233* expression on stromal cells of ectopic and eutopic endometrial in women with EM. The examined women were aged between 23 and 55 yr, had normal menstrual cycles, and had not been given hormones for a few months before sampling. This study demonstrated a reduction in the expression of *miR-223* in the endometrial stromal cells of individuals with EM; the researchers indicated that the upregulation of *miR-223* hindered the production of EMT-related molecules, therefore reducing cell migration and proliferation (31). Furthermore, in a study on the implantation process in female mice, it was shown that increasing the expression of *miR-223-3p* affects the leukemia inhibitory factor receptor signaling pathway. Suppressing *LIF* signaling causes a decrease in fertility; therefore, it can be concluded that the increase in the expression of *miR-223-3p* negatively affects the fertility process. This miRNA is connected to the three prime untranslated region of the *LIF* gene and prevents the expression of this gene (32). Moreover, in research on uterine receptivity in animal cases, it was shown that calcitonin can promote uterine receptivity by raising the expression of *LIF* and lowering the expression of *miR223-3p* via the ERK1/2-mTOR signaling pathway (33). Dexamethasone lowered uterine receptivity during the implantation phase by boosting the expression of *miR223-3p* and diminishing the expression of *LIF* in the endometrial sample of mice (34). Moreover investigating the relationship between electroacupuncture and the expression level of *miR223-3p* on the rate of uterine receptivity in rats, showed that the treatment with electroacupuncture in the uterus of rats by reducing the expression level of *miR223-3p* caused a slight increase in the implantation of blastocytes, resulting in, enhanced uterine receptivity and the occurrence of implantation procedure (35). According to the available evidence, *miR-223* suppresses the metastasis of cervical cancer cells, preventing the process of EMT by augmenting E-cadherin and α -cadherin and reducing the mesenchymal marker vimentin. This shows that *miR-223* might become a novel EM-targeting approach. *miR-223* suppresses the metastasis of human cervical cancer cells by altering EMT (36). The difference in *miR-223-3p* expression level between our study and prior research may be attributed to a variety of factors, such as variations in the cases and control population, climate, race, sampling time, the stage of EM, and the type of sample.

According to the findings of this study, the expression level of *miR-223-3p* and *miR-200a* in the eutopic endometrial tissue of women with EM was significantly lower than in the control group (p < 0.0001).

## 5. Conclusion

Compared to the control group, women with EM exhibited significantly reduced expression levels of *miR-223-3p* and *miR-200a* in the eutopic endometrial tissue. Our findings suggest that these miRNAs, especially *miR-200a* and *miR-223-3p*, are implicated in the disease's pathogenesis, while it is probable that other genes and signaling pathways contribute to the implantation failure associated with this condition. Consequently, by conducting additional research, these miRNAs may be utilized as diagnostic biomarkers for EM.

##  Data Availability

The data supporting the findings of this study are available upon reasonable request from the corresponding author, Naser Shokrzadeh, as they contain information that could compromise the privacy of research participants.

##  Author Contributions

N. Shokrzadeh served as the executive for the experiments and acted as the primary supervisor and study designer. M. Ahmadifard fulfilled the role of second supervisor. Y. Nazari Hagh conducted the experiments and performed the analysis of the study results. S. Abbaszadeh and S. Esmaelzadeh were responsible for examining cases and collecting samples. All authors have approved the final manuscript and assume responsibility for the integrity of the data.

##  Conflict of Interest

The authors declare that there is no conflict of interest.
